# Tunicamycin Depresses P-Glycoprotein Glycosylation Without an Effect on Its Membrane Localization and Drug Efflux Activity in L1210 Cells

**DOI:** 10.3390/ijms12117772

**Published:** 2011-11-10

**Authors:** Mário Šereš, Dana Cholujová, Tatiana Bubenčíkova, Albert Breier, Zdenka Sulová

**Affiliations:** 1 Institute of Molecular Physiology and Genetics, Centre of Excellence of the Slovak Research and Development Agency “BIOMEMBRANES2008”, Slovak Academy of Sciences, Vlárska 5, Bratislava 83334, Slovakia; E-Mails: mario.seres@savba.sk (M.Š.); tatiana.kurucova@savba.sk (T.B.); 2 Cancer Research Institute, Slovak Academy of Sciences, Vlárska 7, Bratislava 83391, Slovakia; E-Mail: dana.cholujova@savba.sk

**Keywords:** P-gp (MDR1), tunicamycin, *N*-glycosylation, L1210

## Abstract

P-glycoprotein (P-gp), also known as ABCB1, is a member of the ABC transporter family of proteins. P-gp is an ATP-dependent drug efflux pump that is localized to the plasma membrane of mammalian cells and confers multidrug resistance in neoplastic cells. P-gp is a 140-kDa polypeptide that is glycosylated to a final molecular weight of 170 kDa. Our experimental model used two variants of L1210 cells in which overexpression of P-gp was achieved: either by adaptation of parental cells (S) to vincristine (R) or by transfection with the human gene encoding P-gp (T). R and T cells were found to differ from S cells in transglycosylation reactions in our recent studies. The effects of tunicamycin on glycosylation, drug efflux activity and cellular localization of P-gp in R and T cells were examined in the present study. Treatment with tunicamycin caused less concentration-dependent cellular damage to R and T cells compared with S cells. Tunicamycin inhibited P-gp *N*-glycosylation in both of the P-gp-positive cells. However, tunicamycin treatment did not alter either the P-gp cellular localization to the plasma membrane or the P-gp transport activity. The present paper brings evidence that independently on the mode of P-gp expression (selection with drugs or transfection with a gene encoding P-gp) in L1210 cells, tunicamycin induces inhibition of *N*-glycosylation of this protein, without altering its function as plasma membrane drug efflux pump.

## 1. Introduction

P-glycoprotein (P-gp), also known as ABCB1, is a member of the ABC transporter family of proteins, and it is an integral protein of the plasma membrane of animal cells [1]. When expressed in neoplastic tissue, P-gp represents a real obstacle for effective chemotherapy of neoplastic diseases, and tissues with increased P-gp are most often observed with the multidrug resistance (MDR) phenotype [2]. The known substrates of this protein represent a large group of unrelated substances, including vincristine, doxorubicin, mitomycin C, actinomycin D, cyclophosphamide and dexamethasone [3]. This protein encoded by the *mdr1* (*abcb1*) gene is first synthesized as a 140-kDa polypeptide precursor that is later glycosylated to a final molecular weight of 170 kDa [4,5]. Each molecule of P-gp contains two nucleotide binding domains with the ABC consensus motif and two transmembrane domains that consist of six α-helical membrane spans [1].

Glycosylation of P-gp occurs on the first extracellular loop, which contains three putative glycosylation sites [6], and glycosylation of P-gp was not found to be necessary for the drug transport activity of P-gp. However, glycosylation of P-gp was shown to be important for proper quality control of P-gp in the endoplasmic reticulum [7] and proper transport of P-gp to the plasma membrane [6]. Tunicamycin is generally known to inhibit the process of protein *N*-glycosylation in the endoplasmic reticulum by blocking the transfer of *N*-acetylglucosamine-1-phosphate from uridine diphosphate-*N*acetyl- glucosamine to dolichol phosphate [8]. However, tunicamycin also induced an elevation of P-gp expression (at both the mRNA and protein levels) and efflux activity in Fao hepatoma cells [9]. This effect was due to endoplasmic reticulum stress and was comparable with the effects of other endoplasmic reticulum stress inducers, such as 2-deoxy glucose [10] and thapsigargin [11]. In contrast, tunicamycin-induced inhibition of total glycoprotein formation, including P-gp, was described for several cell models, and inhibition of glycosylation conferred an increased sensitivity to different drugs [12]. Inhibition of P-gp glycosylation by tunicamycin was associated with increased ubiquitination and subsequent degradation of P-gp [13]. Thus, tunicamycin may induce either an increase or decrease in drug resistance associated with an improvement or impairment of P-gp function, respectively. This dual effect of tunicamycin seems to be due to differences between cell types.

In the present study, we used two variants of L1210 cells that highly express P-gp. These variants were obtained from parental cells (S) either by stepwise adaptation to the drug vincristine (R) [14] or by stable transfection with the human gene encoding P-gp (T) [15]. The MDR phenotype of R cells was associated with an alteration in transglycosylation reactions linked with decreases in UDP-sugars, glycogen and cell surface sialic acid [16]. In addition, R cells differ from S cells in the composition of the cell surface saccharides that are ligands of concanavalin A and the tomato lectin *Lycopersicum esculentum* agglutinin [17]. While concanavalin A interacts more potently with S cells than with R cells, the tomato lectin exhibited the opposite behavior. Both lectins were shown to interact with glycosylated proteins in the plasma membrane other than P-gp [17]. The weak interaction of concanavalin A with P-gp-positive cells is directly related to presence of P-gp in L1210 cells because T cells displayed a similarly reduced interaction compared to S cells [15]. In addition, concanavalin A induced less cell death in R and T cells than in S cells. Therefore, both P-gp-positive L1210 cell variants are also cross-resistant to concanavalin A-induced cell death. Resistance to concanavalin A was found to be due to a defect in the overall biosynthesis of glycoproteins [18]. Alterations in the transglycosylation reaction and consequently in glycoprotein processing were also shown in the P-gp-positive L1210 cell variants [15–17]. Therefore, in the present study, we examined the effect of tunicamycin on P-gp glycosylation, membrane localization and transport activity.

## 2. Results and Discussion

### 2.1. P-gp Expression and Transport Activity in P-gp-Positive L1210 Cell Variants

**Figure 1:**
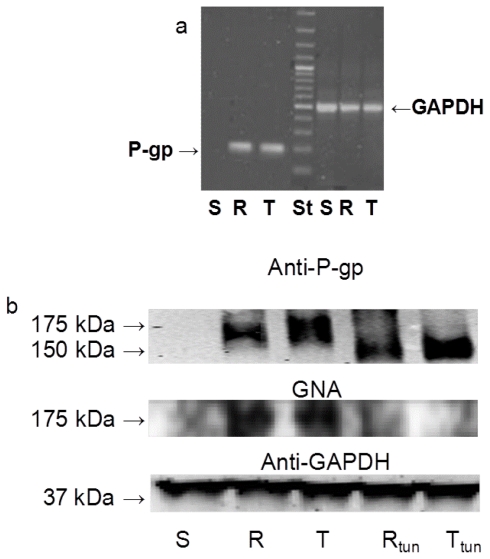
(**a**) Detection of mRNA encoding P-gp using reverse transcription polymerase chain reaction. PCR products obtained from total mRNA isolation from S, R and T cells by reverse transcription and PCR amplification with P-gp-specific primers were subjected to agarose gel electrophoresis (St represents molecular weight standards). PCR products of glyceraldehyde 3-phosphate dehydrogenase (GAPDH) were used as an internal standard. (**b**) Western blot and *Galanthus nivalis* agglutinin (GNA) lectin blot analysis for detection of P-gp. Western blotting for GAPDH was used as an internal control for protein loading. The cells were three passages cultivated in the absence or presence of 0.1 μmol/L tunicamycin. These data are representative of three independent measurements.

**Figure 2:**
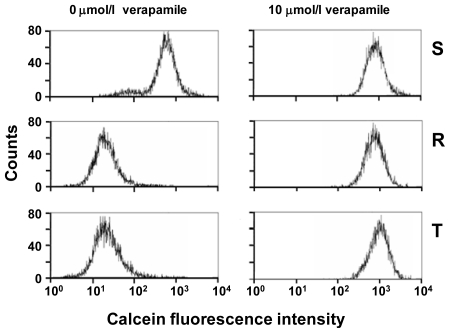
Detection of P-gp function by calcium retention assay in FACS. Data are representative of three independent measurements.

Both P-gp-positive L1210 cell variants (R and T cells) expressed large amounts of mRNA encoding P-gp (Figure 1a), whereas this transcript was barely detectable in S cells. P-gp protein is also detectable in R and T cells, but not in S cells using western blotting with the anti-P-gp antibody c219 (Figure 1b). Western blotting showed a P-gp band with a molecular weight of 170 kDa in R and T cells that indicated a fully glycosylated form [5,7]. Several fluorescent substances are known to be substrates of P-gp and are often used for detection of P-gp transport function directly in intact cells [1]. Calcein/AM, an intracellular calcium indicator, in the esterified form represents a suitable substrate for P-gp, but after intracellular deesterification, it is no longer transportable by P-gp [19,20]. The efflux activity of P-gp protects R and T cells against retention of calcein, while S cells were considerably loaded by this indicator (Figure 2). Verapamil, a blocker of the L-type calcium channel, is also known to be a potent inhibitor of P-gp [21]. This substance is able to block P-gp in R and T cells and allowed loading of these cells by calcein (Figure 2). A similar effect as verapamil could be achieved by the application of cyclosporine A (data not shown), which is known to be another potent inhibitor of P-gp [21]. Thus, overexpression of P-gp in both R and T cells at the levels of mRNA and protein conferred functional efflux activity. This confers strong resistance of R and T cells to P-gp substrates such as vincristine.

### 2.2. The Effect of Tunicamycin on the Viability and Proliferation of S, R and T Cells

**Figure 3:**
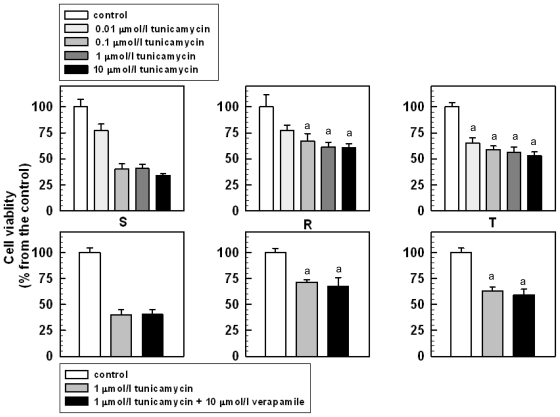
The effect of tunicamycin on the viability of S, R and T cells. Data represent the mean ± SD of six independent measurements. The a indicates a statistically significant difference from the corresponding value obtained for S cells with *p* < 0.01.

Both of the P-gp-positive L1210 cell variants are slightly less sensitive to tunicamycin than the parental S cells. Tunicamycin in the range of 0.01–10.00 μmol/L induced concentration-dependent depression of proliferation that could be monitored by the MTT assay (Figure 3). While proliferation of S cells is blocked to 30% of the value of the untreated control at the highest applied tunicamycin concentration, more than 50% of R and T cells were viable under the same condition.

The lower sensitivity of the P-gp-positive L1210 cell variants to tunicamycin could be directly related to P-gp expression because is independent of the method of P-gp expression (*i.e.*, adaptation to vincristine or transfection with the human gene encoding P-gp). However, the decrease in tunicamycin sensitivity of the P-gp-positive R and T cells is unrelated to P-gp transport activity because verapamil, a potent inhibitor of this activity, induced only non-significant effects on the viability of tunicamycin-treated R and T cells (Figure 3).

The main difference between the P-gp-negative S cells and the P-gp-positive R and T cells was obtained by repeated cultivation of cells in the presence of 0.1 μmol/L tunicamycin. While R and T cells showed no growth effect in response to repeated cultivation in treatment medium, S cells failed to grow after the third passage of cultivation (Supplementary Figure Ia). The viability of R and T cells after repeated cultivations with tunicamycin measured by fluorescein diacetate and propidium iodide was not altered (Supplementary Figure Ib). The decrease of fluorescein diacetate labeling was visible in S cells after the third passage of cultivation in the presence of tunicamycin. The anti-proliferative effect of tunicamycin on L1210 cells was associated with a decrease in the incorporation of sugar moieties onto cellular glycoproteins [22]. Morin and colleagues found that the treatment of cells with tunicamycin resulted in an increase of intracellular pool of UDP-*N*-acetylglucosamine that was associated with distentions of the endoplasmic reticulum and the nuclear membranes. Similar ultrastructural changes and increases in the intracellular pools of UDP-sugars were observed in L1210 cells exposed to 5 mmol/L d-glucosamine, which suggests that the antiproliferative effects of tunicamycin may be related to the accumulation of one or more of the nucleotide-sugar precursors of asparagine-linked glycoprotein biosynthesis in the endoplasmic reticulum [22]. We have previously described a strong decrease of UDP-sugars in P-gp-positive L1210 cells [16]. Therefore, the resistance of P-gp-positive L1210 cells to tunicamycin may be due to the decreased levels of UDP-sugars in these cells.

### 2.3 The Effect of Tunicamycin on Glycosylation and Membrane Localization of P-gp

**Figure 4:**
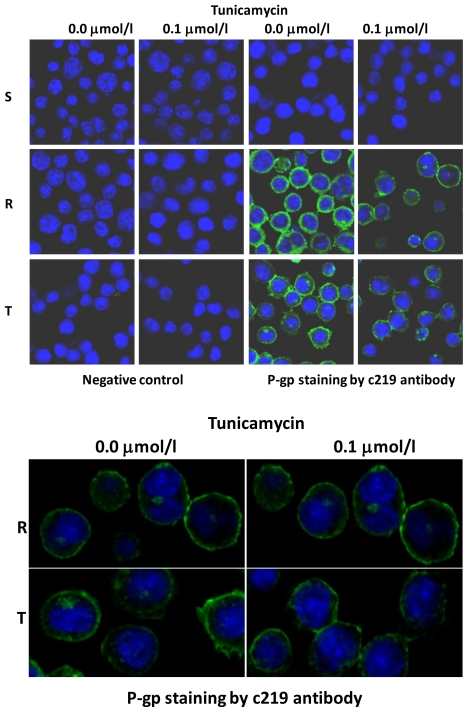
(**a**) Visualization by immunofluorescence confocal microscopy of the localization of P-gp in cells cultivated for three passages in the absence or presence of tunicamycin. Nuclei were stained using 4′,6-diamidino-2-phenylindole (blue). P-gp was detected using the c219 anti-P-gp antibody and a FITC-conjugated anti-mouse secondary antibody (green). In the negative control, the c219 antibody was omitted, and the cells were incubated with a FITC-conjugated anti-mouse secondary antibody alone. These data are representative of three independent measurements. (**b**) Cells from the same experiments in (**a**) at a higher magnification.

P-gp could be detected in crude membrane fractions using western blotting as a 170-kDa protein band, which indicated a fully glycosylated mature glycoprotein (Figure 1b). When R and T cells were repeatedly cultivated in the presence of tunicamycin, P-gp was detected using western blotting with the same antibody as 150-kDa protein bands, suggesting that the protein was non-glycosylated. This is shown in Figure 1b after three passages of repeated cultivation in the presence of tunicamycin. Similar results were obtained when cells were cultivated in the presence of tunicamycin for one, six or twenty-four passages of cultivation (the presence of non-glycosylated P-gp in R and T cells after 24 passages is documented in Supplementary Figure II). We have previously observed the same 150-kDa non-glycosylated P-gp when thapsigargin, another inducer of endoplasmic reticulum stress, was applied [23]. The sugar chains attached to P-gp were recently found to interact with *Galanthus nivalis* agglutinin (GNA) and *Sambucus nigra* agglutinin (SNA) [4]. Crude membranes isolated from R and T cells contained a protein band of 170 kDa that could be stained by GNA using a lectin blot (Figure 1b). There was no protein band observed using lectin blot with GNA in the crude membrane fraction isolated from S cells. The protein bands detected using the GNA-specific lectin blot in R and T cells were diminished when these cells were treated with tunicamycin. In another set of experiments, we observed similar results using an SNA-specific lectin blot (data not shown). Taken together, these results illustrate that tunicamycin potently inhibited P-gp glycosylation because only non-glycosylated P-gp is present in both P-gp-positive cell variants after treatment with this antibiotic. Because we were using crude membrane preparations that include all cell membrane structures in this western blot analysis, the localization of the non-glycosylated P-gp after treatment with tunicamycin was unknown. Therefore, we examined the cellular localization of P-gp after tunicamycin treatment using immunofluorescence confocal microscopy with the c219 anti-P-gp antibody and a FITC-conjugated anti-mouse secondary antibody. Both P-gp-positive variants of L1210 cells showed the P-gp protein localized to the surface structures of the cells. In contrast, P-gp could not be detected in S cells, similar to our results from western blot analysis. After R and T cells had been cultivated in the presence of tunicamycin for three passages, which resulted in the non-glycosylated 150-kDa form of P-gp (Figure 1b), the localization of P-gp was still detected on surface structures of the cells, similar to untreated cells (Figure 4). Similar results were obtained when cells were cultivated in the presence of tunicamycin for one, six or twenty-four passages (data not shown). Thus, the prevention of P-gp *N*-glycosylation by tunicamycin did not alter its cell localization.

The inhibition of *N*-glycosylation by tunicamycin was shown to induce an increase in P-gp ubiquitination and proteasomal degradation in a P-gp-positive MCF7 cell variant [13]. Consistent with this finding, treatment with tunicamycin caused a reduction of the P-gp-mediated MDR phenotype [24]. Degradation by the proteasome represents the final step for incorrectly synthesized proteins, including P-gp, during quality control in endoplasmic reticulum [7]. However, the application of tunicamycin to human MDR KB-C1 cells induced a hypersensitivity to 2-deoxyglucose due to an alteration of the function of the glucose transporter GLUT1, which did not influence P-gp transport activity [25]. Therefore, in some cases after treatment with tunicamycin, non-glycosylated P-gp could escape from the proteasomal degradation pathway. Both P-gp-positive L1210 cell variants used in the current study represent examples of the latter behavior.

### 2.4. Effect of Tunicamycin on P-gp Transport Activity

**Figure 5:**
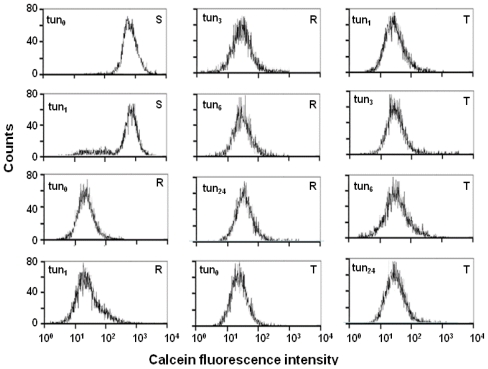
Calcein/AM retention assay of the influence of tunicamycin on P-gp transport function. Cells (S, R and T) were cultivated in the absence (tun_0_) or the presence of one (tun_1_), three (tun_3_), six (tun_6_) or twenty-four passages (tun_24_) of 0.1 μmol/L tunicamycin treatment, and then the calcium/AM assay was performed. These data are representative of three independent measurements.

As previously described above, R and T cells could be repeatedly cultivated in the presence of 0.1 μmol/L tunicamycin. In contrast, S cells stopped proliferating after the third passage of cultivation in this condition. Therefore, we used S cells cultivated in the absence or presence of a single passage of tunicamycin treatment in a calcein retention assay. The R and T cells used in the calcein retention assay were cultivated in the absence or presence of one, three, six or twenty-four passages of tunicamycin treatment. S cells exhibited retention of calcein independent of the presence or absence of tunicamycin (Figure 5). In contrast, the retention of calcein was reduced in R and T cells due to the overexpression of P-gp, and the efflux activity of P-gp was not affected by the presence of tunicamycin during repeating cultivation. Therefore, after tunicamycin treatment, the non-glycosylated P-gp (Figure 1b) that is incorporated into the plasma membrane (Figure 4) retains the ability to transport calcein out of the cell (Figure 5). Calcein could be retained in R and T cells treated repeatedly with tunicamycin by verapamil (as demonstrated in cells after 24 passages in medium with tunicamycin in Supplementary Figure III). This finding demonstrates that P-gp transport function was not altered in the R and T cells by the inhibition of *N*-glycosylation after tunicamycin treatment. Thus, sufficient levels of P-gp are accurately incorporated into the plasma membrane to fully develop the MDR phenotype in the R and T cells treated with tunicamycin, even though the *N*-glycosylation of P-gp is strongly inhibited by this treatment. Consistent with this hypothesis, tunicamycin treatment of R and T cells failed to alter the resistance to vincristine, another known P-gp substrate (Supplementary Figure III). This resistance to vincristine could be reversed by verapamil. In P-gp-negative HL60/AR cells, the MDR phenotype was found to be due to hypoglycosylated membrane proteins [26]. A similar pattern of hypoglycosylated membrane glycoproteins was observed in drug-sensitive HL60 cells after treatment with tunicamycin, and this antibiotic altered the drug sensitivity of the treated HL60 cells. The question of whether tunicamycin caused any additional alteration of the drug sensitivity in R and T cells due to hypoglycosylated membrane proteins other than P-gp would be of interest for future studies.

## 3. Experimental Section

### 3.1. Cell Culture Conditions

The following three L1210 cell variants were used in this study: (i) S—drug-sensitive parental cells; (ii) R—P-gp-positive drug-resistant cells that overexpress P-gp after selection with vincristine (VCR, from Gedeon Richter Co., Hungary) [14]; and (iii) T—P-gp-positive drug-resistant cells that overexpress P-gp following stable transfection with the P-gp gene [15] using the Addgene plasmid 10957 (pHaMDRwt), a retrovirus encoding the full-length P-gp cDNA [27]. The cells (S, R and T; inoculums 1 × 10^6^ cells) were cultured in 4 mL RPMI 1640 media with l-glutamine (1 mg/mL), 4% fetal bovine serum and 1 μg/mL gentamycin (all purchased from Gibco, USA) in a humidified atmosphere with 5% CO_2_ and air at 37 °C for 48 h in the absence or presence (0.01–10 μmol/L) of tunicamycin. This procedure was termed as passage. R cells were cultured for two passages without VCR prior to the experiments.

### 3.2. Detection of the P-gp mRNA

The total mRNA was extracted from S, R and T cells using the RNA-solvent reagent concentrate R6830-02IN (OMEGA Bio-Tek, USA). mRNA was reverse-transcribed to cDNA using a First Strand cDNA Synthesis Kit (Novagen, USA). Both mRNA extraction and cDNA synthesis were carried out according to protocols recommended by Novagen. PCR reactions were performed using the Nova Taq PCR Master Mix (Novagen, USA). The following PCR primers were used: the *mdr1* gene, 5′-CCC ATC ATT GCA ATA GCA GG-3′ and 5′-GTT CAA ACT TCT GCT CCT GA-3′, which yielded a 167-bp product, and the *gapdh* gene as an internal control, 5′-TAT GTC GTG GAG TCT ACT GGT GTC-3′ and 5′-GTC ATC ATA CTT GGC AGG TTT CTC-3′, which yielded a 453-bp product. PCR reactions were carried out using 30 cycles of a 1 min denaturation step at 94 °C, a 1 min annealing step at 57 °C (for GAPDH) or at 55 °C (for *mdr1*), and a 2 min extension step at 72 °C according to the protocol recommended by Novagen. The PCR products were separated using a 1.7% agarose gel (Invitrogen) and visualized using ethidium bromide with a Typhoon 9210 imager (GE Healthcare, USA, formerly Amersham Biosciences).

### 3.3. Examination of P-gp Function Using Calcein/AM Assay

After cultivation, S, R and T cells (5 × 10^5^) were washed twice in PBS containing 0.1% bovine serum albumin and were then resuspended in 500 μL of the same buffer. Calcein/AM (Sigma-Aldrich, final concentration of 0.1 μmol/L) and propidium iodide (final concentration of 0.9 μmol/L) were added directly to the buffer, and the samples were incubated for 20 min at 37 °C. After incubation, the cells were washed twice in ice cold PBS. Fluorescence was measured using the Coulter Epics Altra flow cytometer (USA)

### 3.4. The Effect of Tunicamycin on S, R and T Cell Proliferation

Cells (5 × 10^4^ cells in each well) were cultured either with or without tunicamycin (concentration range 0.01–10 μmol/L) and verapamil (10 μmol/L) in 96-well culture plates. Verapamil and tunicamycin (obtained from Sigma Aldrich USA) were added directly to the culture media. After 48 h, cell viability was assayed using the MTT test [28], which was performed by adding MTT ([3-(4,5-dimethyldiazol-2- yl)-2,5 diphenyl tetrazolium bromide]) at a final concentration of 0.25 mg/mL to each well. The cells were incubated with MTT for 2 h. Then the plates were centrifuged for 15 min (2500 rpm), and the cell sediment was extracted using dimethyl sulfoxide. The absorbance at 540 nm was measured using a Universal Microplate Spectrophotometer mQuant (BioTek Instruments, Inc. USA). Statistical significance was analyzed using the unpaired Student’s *t*-test.

### 3.5. Examination of the Effect of Tunicamycin on P-gp Levels in S, R and T Cells Using Western Blot and GNA Blot Analysis

After incubation, the cells were harvested, and crude membrane fractions were prepared with a ProteomeExtract Subcellular Proteome Extraction Kit (Calbiochem) according to the manufacturer’s instructions. Proteins from the samples were separated by sodium dodecyl sulfate polyacrylamide electrophoresis (SDS-PAGE) using 8% polyacrylamide gels [29]. Proteins were then transferred by electroblotting to a nitrocellulose membrane [30]. P-gp was detected using the c219 anti-P-gp monoclonal antibody (Calbiochem, USA). A secondary anti-mouse antibody conjugated with horseradish peroxidase (GE Healthcare, USA) was used for detection. To provide an internal control for protein loading, rabbit polyclonal antibodies against GAPDH (Santa Cruz Biotechnology, USA), were used as primary antibodies, and goat anti-rabbit IgG conjugated with horseradish peroxidase (Santa Cruz Biotechnology, USA) served as a secondary antibody. Protein bands were visualized using the ECL detection system (GE Healthcare) and the Kodak (USA) CF 440 imaging system. For GNA-specific lectin blots, proteins were visualized with methods similar to those for western blotting, using a biotinylated-GNA and streptavidin conjugated with peroxidase (Sigma-Aldrich USA).

### 3.6. Visualization of the P-gp in S, R and T Cells Using Immunofluorescence Confocal Microscopy

After cultivation, the cells were washed and resuspended in PBS, and then the cells were transferred onto poly-l-lysine cover glasses (Menzel Glaser, Germany). The bound cells were washed twice in PBS and then fixed with methanol at −20 °C for 20 min after fixation, the cells were washed in PBS and then blocked by incubation with 1% BSA in PBS for 1 h at 37 °C. Next, the cells were incubated with the c219 anti-P-gp antibody for 1 h at 37 °C in PBS containing 1% BSA. After the primary antibody incubation, the cells were washed twice in PBS containing 1% BSA. Then the cells were incubated with FITC-conjugated goat anti-mouse antibody (Calbiochem, USA) in PBS containing 1% BSA for 1 h at 37 °C and then washed twice in PBS containing 1% BSA. The immunofluorescently labeled cells were also labeled with 10 mg/L of 4′-6-diamidino-2-phenylindole (DAPI, Sigma USA) in PBS to visualize the nuclei [31]. Finally, the coverslips were mounted onto slides with a mounting medium (80% glycerol) and analyzed using a confocal laser scanning microscope (LSM 510 META Carl Zeiss).

## 4. Conclusions

Tunicamycin effectively blocked *N*-glycosylation of P-gp in both of the P-gp-positive cell variants of L1210 cells analyzed (R and T cells) because only the non-glycosylated 150 kDa P-gp could be detected using western blotting after cultivation in the presence of 0.1 μmol/L of this antibiotic. In addition, the P-gp in tunicamycin-treated R and T cells was undetectable using a GNA-specific lectin blot. However, inhibition of *N*-glycosylation of P-gp did not alter its plasma membrane localization or drug efflux activity. Thus it could be stated that independently on the mode of P-gp expression (selection with drugs or transfection with gene encoding P-gp) in L1210 cells, tunicamycin induces inhibition of *N*-glycosylation of this protein without altering its function as plasma membrane drug efflux pump.

## Supplementary Material
