# Supplementary material for: Tunicamycin Depresses P-Glycoprotein Glycosylation Without an Effect on Its Membrane Localization and Drug Efflux Activity in L1210 Cells
Source: Int J Mol Sci. 2011 Nov 10;12(11):7772–84. doi: 10.3390/ijms12117772 (PMC3233437; doi:10.3390/ijms12117772)
Supplement: Supplementary file 1 [file ijms-12-07772-s001.pdf]

Supplementary Information

Mário Šereš [1], Dana Cholujová [2], Tatiana Bubenčíkova [1], Albert Breier [1,]* and Zdenka Sulová [1,]*

[1] Institute of Molecular Physiology and Genetics, Centre of Excellence of the Slovak Research and Development Agency "BIOMEMBRANES2008", Slovak Academy of Sciences, Vlárska 5, Bratislava 83334, Slovakia; E-Mails: mario.seres@savba.sk (M.S.); tatiana.kurucova@savba.sk (T.B.)

[2] Cancer Research Institute, Slovak Academy of Sciences, Vlárska 7, Bratislava 833 91, Slovakia; E-Mail: dana.cholujova@savba.sk

* Author to whom correspondence should be addressed; E-Mail: Breier@up.upsav.sk (A.B.); zdena.sulova@savba.sk (Z.S.); Tel.: +421-903-472-606 (A.B.); +421-903-246-360 (Z.S.); Fax: +421-2-5477-3666 (A.B.); +421-2-5477-3666 (Z.S.).

Received: 25 September 2011; in revised form: 20 October 2011 / Accepted: 3 November 2011/ Published: 10 November 2011

Abstract: P-glycoprotein (P-gp), also known as ABCB1, is a member of the ABC transporter family of proteins. P-gp is an ATP-dependent drug efflux pump that is localized to the plasma membrane of mammalian cells and confers multidrug resistance in neoplastic cells. P-gp is a 140-kDa polypeptide that is glycosylated to a final molecular weight of 170 kDa. Our experimental model used two variants of L1210 cells in which overexpression of P-gp was achieved: either by adaptation of parental cells (S) to vincristine (R) or by transfection with the human gene encoding P-gp (T). R and T cells were found to differ from S cells in transglycosylation reactions in our recent studies. The effects of tunicamycin on glycosylation, drug efflux activity and cellular localization of P-gp in R and T cells were examined in the present study. Treatment with tunicamycin caused less concentration-dependent cellular damage to R and T cells compared with S cells. Tunicamycin inhibited P-gp *N*-glycosylation in both of the P-gp-positive cells. However, tunicamycin treatment did not alter either the P-gp cellular localization to the plasma membrane or the P-gp transport activity. The present paper brings evidence that independently on the mode of P-gp expression (selection with drugs or transfection with a gene encoding P-gp) in L1210 cells, tunicamycin induces inhibition of *N*-glycosylation of this protein, without altering its function as plasma membrane drug efflux pump.

Keywords: P-gp (MDR1); tunicamycin; *N*-glycosylation; L1210

Figure I. The effect of repeated cultivation in tunicamycin on the proliferation and viability of S, R and T cells. (**a**) Detection of cell number after 1, 3, 6 and 24 passages in cultivation medium containing 0.1 µmol/L tunicamycin. Cultivation of cells under the same conditions but without tunicamycin (see chapter 3.1) produced the following numbers of cells 2.9 ± 0.2 × 10^6, 2.7 ± 0.1 × 10^6 and 2.8 ± 0.2 × 10^6 for S, R and T cells, respectively. Data represent the mean ± Sd of six independent measurements. White columns—S cells; grey columns—R cells; black columns—T cells. (**b**) Viability of cells after repeated cultivation in the presence of 0.1 µmol/L tunicamycin. After the passage cells were washed twice in PBS and then incubated for 30 min. in PBS containing fluorescein diacetate (final concentration 0.5 mg/L) in a humidified atmosphere with 5% CO_2 and air at 37 °C. After incubation cells were washed and resuspended in an ice cold PBS and propidium iodide (final concentration 50 mg/L) was added. Fluorescence was measured using the Coulter Epics Altra flow cytometer (USA). These data are representative of three independent measurements.

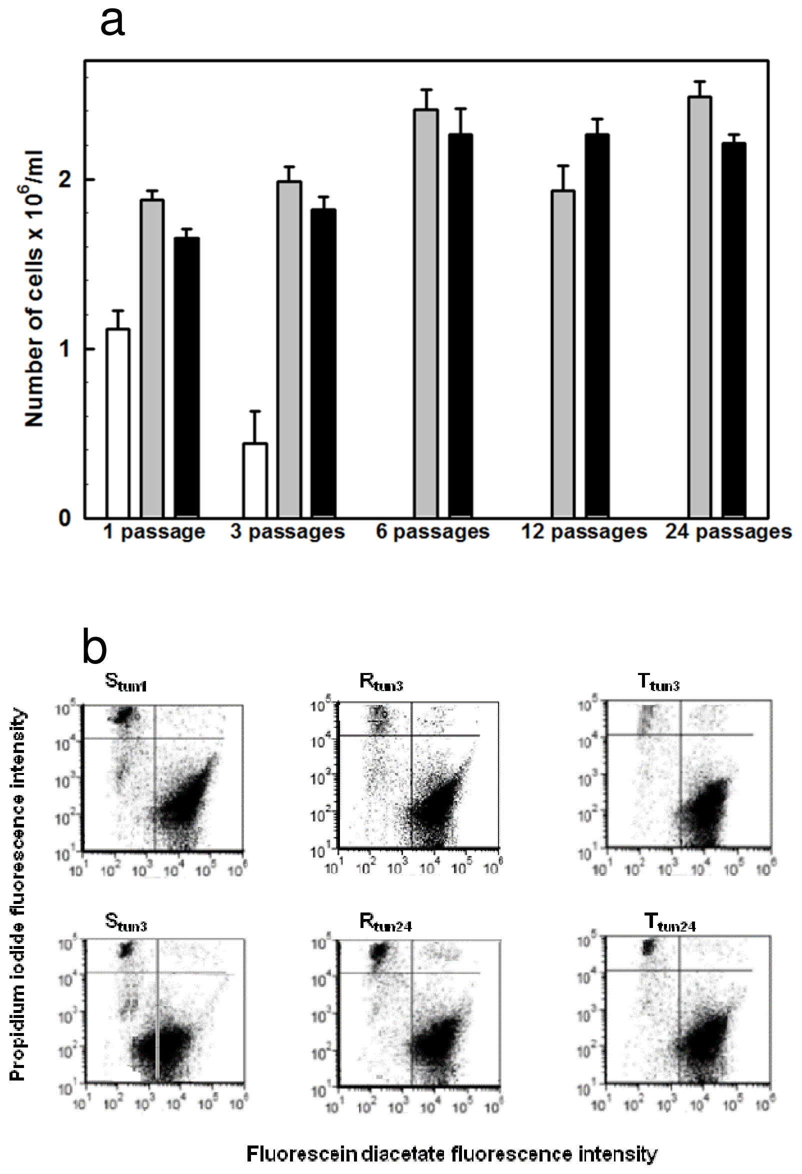

Figure II. Western blot analysis for detection of P-gp. Western blotting for GAPDH was used as an internal control for protein loading. The cells were cultivated during twenty four passages in the absence or presence of 0.1 μmol/L tunicamycin. These data are representative of three independent measurements.

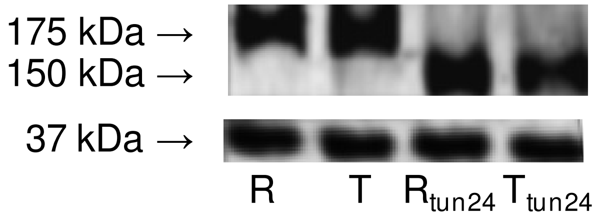

Figure III. (**a**) Reversal of vincristine resistance of R and T cells with verapamil. Both drugs were added directly to 200 μL of cultivation medium and cells (inoculums 5 × 10^4) were cultivated under standard conditions in 96 wells culture plates. Cell proliferation was estimated by MTT test. Data document a strong vincristine resistance because IC_{50} value for S cells is 0.01 mg/L (Polekova *et al.* 1992). Data represent the mean ± S_d of six independent measurements. (**b**) Detection of P-gp function and its verapamil inhibition by calcium retention assay in R and T cells cultivated during 24 passages in the absence or in the presence of tunicamycin. Data are representative of three independent measurements.

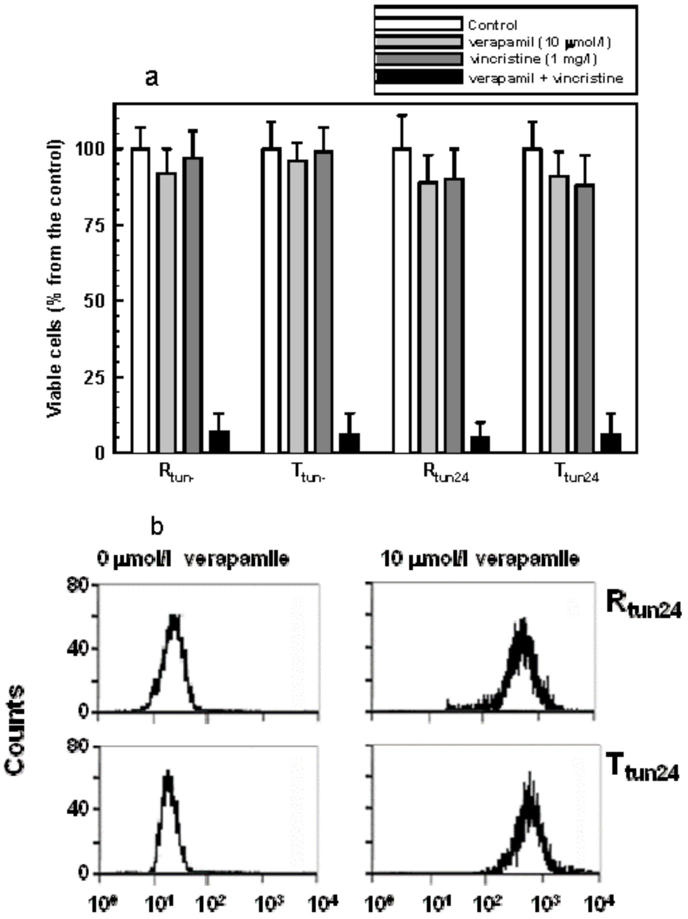

© 2011 by the authors; licensee MDPI, Basel, Switzerland. This article is an open access article distributed under the terms and conditions of the Creative Commons Attribution license (http://creativecommons.org/licenses/by/3.0/).
